# New-onset atrial fibrillation can be falsely associated with increased length of stay in ICU due to immortal time bias

**DOI:** 10.1186/s13054-020-2757-2

**Published:** 2020-02-06

**Authors:** Yifang Lu, Tenggao Chen

**Affiliations:** 1 Department of oncology, Affiliated Dongyang Hospital of Wenzhou Medical University, Dongyang, Zhejiang People’s Republic of China; 2 Department of colorectal surgery, Affiliated Dongyang Hospital of Wenzhou Medical University, Dongyang, Zhejiang People’s Republic of China

Dear editor,

In a recent study published in *Critical Care*, Fernando SM and colleagues investigated the impact of new-onset atrial fibrillation (NOAF) on clinical outcomes in critically ill patients [1]. They performed univariate analysis and found that the length of stays (LOS) in ICU and hospital was both longer in the NOAF group versus non-NOAF group. They then concluded that NOAF was associated with increased LOS in ICU and increased total costs. While the conclusion appeared intuitive and statistically sound, it could be the result of immortal time bias. Immortal time is a span of cohort follow-up during which, because of exposure definition, the outcome under study could not occur [2]. The NOAF can happen at any time during ICU stay and patients live longer in the ICU can have more chance to report NOAF. For example, a patient can have NOAF on day 4 and the outcome such as ICU discharge or death cannot happen before day 4. In this situation, the period from days 1 to 3 are considered as immortal time because if the outcome happens during the period, the patient cannot experience NOAF. The immortal time is incorrectly attributed to the exposure of NOAF, but actually, the NOAF do not contribute to the survival time. The same applies to the mortality outcome. The authors used binary logistic regression model to adjust for confounding effect and found there was no independent association of NOAF and mortality [3]. The truth could be that NOAF is associated with increased mortality risk, but since patients who lived longer can have more chances to experience NOAF, the neural effect reported in the paper was actually the result of the true adverse effect and the bias towards beneficial effect. Potential solutions to control for the immortal time bias are as follows: (1) perform analysis by restricting to patients who had NOAF on day 1 and compare to those without NOAF, (2) consider the time of NOAF and include NOAF as time-varying covariate in the Cox proportional hazard model [4], and (3) perform time-dependent propensity score matching by including covariates that can influence the onset of NOAF [5].

## Data Availability

No data for the work
