# Supplementary material for: New-onset atrial fibrillation can be falsely associated with increased length of stay in ICU due to immortal time bias
Source: Crit Care. 2020 Feb 6;24:40. doi: 10.1186/s13054-020-2757-2 (PMC7006403; doi:10.1186/s13054-020-2757-2)
Supplement: Related texts

# Reply: Authors’ response

**Authors:** Shannon M. Fernando, Rebecca Mathew, Benjamin Hibbert, Bram Rochwerg, Laveena Munshi, Allan J. Walkey, Morten Hylander Møller, Trevor Simard, Pietro Di Santo, F. Daniel Ramirez, Peter Tanuseputro, Kwadwo Kyeremanteng

The authors would like to thank Drs. Lu and Chen for their comments on our recent article related to outcomes and costs associated with new-onset atrial fibrillation (NOAF) in critically ill adults [1]. Drs. Lu and Chen suggest caution in the interpretation of our study results, particularly as they relate to the length of stay and costs, due to the possibility of immortal time bias. Indeed, the use of time-dependent covariates (such as NOAF) has the potential to confound multivariate models [2]. However, as seen in our original study, the majority of patients who developed NOAF did so on the first day of hospital admission, consistent with previous studies in this population [6, 7]. As suggested by Drs. Lu and Chen, we conducted sensitivity analyses of our data, restricting the NOAF population to only those who developed NOAF on the first day of hospital admission (*n* = 962, 62.4% of the original NOAF population). Indeed, our original results persist, with patients with developing NOAF on the first day of admission still experiencing prolonged hospital stay, as compared to patients who did not develop NOAF (Table 1). We similarly repeated our generalized linear model restricted to this select population, in order to determine if NOAF was still associated with total hospital costs (Table 2). We found that NOAF was indeed a predictor of total hospital costs, even when only considering patients who developed NOAF on the first day of admission. These findings support the notion that our results were not markedly influenced by immortal time bias.

**Table 1:** Outcomes of ICU patients with new-onset atrial fibrillation (NOAF) on the first day of admission and those without NOAF

Characteristic	New-onset atrial fibrillation on day 1 (*n* = 962)	No atrial fibrillation (*n* = 13,473)	Adjusted odds ratio^c^ (95% CI)	*P* value
In-hospital mortality, *n* (%)	367 (38.1)	4034 (29.9)	1.03 (0.95–1.11)	0.34
Persistent atrial fibrillation, *n* (%)^b^	238 (24.7)	–		
ICU length of stay, days, median (IQR)	7 (2–14)	6 (2–9)		< 0.01
Hospital length of stay, days, median (IQR)	14 (6–27)	12 (4–25)		< 0.001
Ventilator-free days, median (IQR)	6 (2–8)	6 (4–10)		0.08

*Abbreviations: ICU* intensive care unit, *IQR* interquartile range

^a^Only includes patients surviving to discharge

^b^Defined as the presence of any atrial fibrillation following 24 h of treatment

^c^Ratio of NOAF to patients with no atrial fibrillation

**Table 2:** Generalized linear model with gamma distribution and log link for the total cost for patients with new-onset atrial fibrillation (NOAF) on the first day or admission and those without NOAF (*n* = 14,435)

Variable	Cost ratio	95% CI	*P* value
Age (per 5 years)	0.96	0.90–1.03	0.07
Male gender	1.01	0.88–1.12	0.17
New-onset atrial fibrillation	1.08	1.02–1.19	< 0.01
MODS (per 1 point)	0.96	0.91–0.98	< 0.01
Comorbidities			
Congestive heart failure	0.96	0.87–1.06	0.35
Peripheral vascular disease	1.05	0.97–1.13	0.22
Hypertension	1.02	0.92–1.09	0.66
Chronic obstructive pulmonary disease	0.95	0.88–1.12	0.59
Diabetes mellitus	1.02	0.92–1.11	0.47
Chronic kidney disease	0.98	0.81–1.21	0.42
Liver disease	0.91	0.77–1.08	0.31
Alcohol misuse	1.02	0.95–1.08	0.77
Elixhauser comorbidity score (per 1 point)	0.99	0.90–1.07	0.53
No CPR directive at ICU admission	0.82	0.70–0.92	< 0.001
Location prior to ICU admission			
Emergency department	Ref		
Hospital wards	1.04	0.87–1.20	0.51
Operating room	1.09	0.99–1.29	0.06
Peripheral hospital	0.93	0.79–1.06	0.11
Most responsible diagnosis			
Other	Ref		
Infection/sepsis	1.06	0.89–1.20	0.45
Respiratory failure	1.91	1.29–2.41	< 0.001
Trauma	1.49	1.15–1.88	< 0.001
Malignancy	0.94	0.79–1.10	0.38
Spontaneous intracranial hemorrhage	2.19	1.71–2.60	< 0.001
Stroke	1.14	1.01–1.28	0.04
Overdose/poisoning	0.88	0.80–0.96	< 0.01
Renal failure	0.83	0.66–0.93	< 0.001
Gastrointestinal bleeding	1.12	0.97–1.20	0.10
Congestive heart failure	1.06	0.95–1.15	0.39
Cardiac arrest	1.13	0.89–1.23	0.68
Seizures/status epilepticus	1.19	1.01–1.40	0.03
Diabetic ketoacidosis	0.73	0.61–0.83	< 0.001
In-hospital death	0.63	0.56–0.69	< 0.001
Length of stay (per 1 day)	1.03	1.02–1.05	< 0.01
Invasive mechanical ventilation	1.25	1.16–1.35	< 0.001
Renal replacement therapy	1.09	1.02–1.14	< 0.01

*Abbreviations: MODS* Multiple Organ Dysfunction Score, *ICU* intensive care unit, *CI* confidence interval, *CPR* cardiopulmonary resuscitation

Finally, we would like to clarify that total hospital costs are not inferred as a multiple of hospital length of stay, as Drs. Lu and Chen suggest. In fact, all direct costs are linked directly to the patient identification number, and while they may be associated with length of stay (e.g., a person who stays in hospital for a longer length of time is likely to have increased testing and therapies, translating to higher costs), they are not obtained through a multiplication of length of stay, but rather reflect the direct resource use for each individual patient [8].
